# Characteristics and outcomes of the drug patent linkage system in China

**DOI:** 10.1186/s12992-024-01035-x

**Published:** 2024-04-15

**Authors:** Xue-Fang Yao

**Affiliations:** School of Health Economics and Management, Nanjing University of Chinese Medicine, Nanjing, Jiangsu Province China

**Keywords:** Patent linkage, Patent listing, Patent certification, Access to medicines, China

## Abstract

**Background:**

On July 4, 2021, China officially introduced the drug patent linkage system, which has made more localized adjustments than have similar systems in the US and South Korea. This study describes the characteristics and outcomes of China’s patent linkage system.

**Methods:**

For this study, we used the database of China’s patent information registration platform for marketed drugs to capture all listed patents and patent certifications from June 25, 2021, to June 30, 2023. We used descriptive statistics for the above data to assess the impact of patent linkage on branded drug manufacturers, generic drug manufacturers, and the public’s access to medicines.

**Results:**

During the study period, the patents of 632 branded drugs were listed, and 5058 ANDAs submitted patent certifications to the Registration Platform. Of these 632 branded drugs, 462 (73.1%) drugs were approved before the year of patent registration, and the average number of listed patents per drug was 1.8, with a standard deviation of 1.4. However, of these 5058 ANDAs, P1 certifications accounted for 85.1%, and P3 and P4 certifications accounted for 16% combined. In addition, according to the detailed statistics of P2 certifications, we found that the proportion of patent invalidation cases was 46.4%. The remaining validity of the patents corresponding to P3 certifications was longer, with a median value of 17 months, and the IQR was 10-30.75, ranging from − 2 to 204 months.

**Conclusions:**

China’s patent linkage aims to promote the balance of multiple interests —innovation, imitation and public health—and has its own system characteristics. Patent listing and patent certification are the key indicators reflecting the implementation effect of the system. From the perspective of system outcomes, ANDAs have been connected to the patent linkage system in an orderly manner, but the growth of patent challenges is not obvious. Moreover, manufacturers of foreign branded drugs that have not yet entered the Chinese market need to pay more attention to the role of patent listing.

**Supplementary Information:**

The online version contains supplementary material available at 10.1186/s12992-024-01035-x.

## Background

The drug patent linkage system originated in the U.S. (1984) and was subsequently introduced in Canada (1993), Australia (2004), Singapore (2004), South Korea (2015) and other regions; among these regions, the implementation of regional or bilateral trade agreements was considered an important contributor [1]. The introduction of a patent linkage system in China has also been influenced by the China‒US Economic and Trade Agreement (2020), in which the “Intellectual property rights related to pharmaceuticals” chapter contains a clause on an effective mechanism for the early settlement of patent disputes [2]. However, the system was introduced mostly because the industry believes that patent linkages can promote the balance of multiple interests, such as innovation, imitation and public health [3]. In October 2020, China’s newly revised Patent Law established a patent linkage at the legislative level for the first time [4]. Subsequently, on July 4, 2021, the China National Intellectual Property Administration (CNIPA) and National Medical Products Administration (NMPA) jointly issued the ***Measures for the Implementation of the Mechanism for Early Settlement of Drug Patent Disputes (interim)***, marking the official implementation of the patent linkage [5]. To complete the implementation of the system, China’s Patent Information Registration Platform for Marketed Drugs (hereinafter referred to as the Registration Platform) was officially put into operation on the same day [6].

A patent linkage is essentially a system that links the marketing approval of generic drugs to the status of the patent(s) corresponding to the branded drugs to identify and resolve possible patent infringement problems before the approval of generic drugs [7]. In terms of system content, China’s patent linkage system included five parts: patent listing (registers only those drugs marketed in China), patent certification (four types from P1 to P4), procedure linkage of patent challenge (including notification and stay period), approval of generic drug application (also known as an abbreviated new drug application (ANDA)), and first generic exclusivity. The patent list and patent certification are published on the Registration Platform. This study compares this system with those of the U.S. (the system is more mature) and South Korea ( a newly introduced system) (see Additional file 1 for a detailed comparison). However, China has its own characteristics in the system setting, which are listed below.

### Patent listing

A patent listing is the key point for safeguarding the rights and interests of innovators in the patent linkage system. In contrast, the main differences between patent listings in the US and South Korea and those in China are reflected in two aspects. First, the patents that may be listed, including drug substance, composition, formulation, and pharmaceutical use [8], are basically the same in the US and South Korea, but China does not include formulation in its patent listings. Second, for the management of patent lists, South Korea’s Ministry of Food and Drug Safety (MFDS) has the right to review and modify validity, while China’s Center for Drug Evaluation (CDE) has authority only over administrative management. Whether a patent listing is appropriate is the responsibility of the patentee him- or herself, which is similar to the system in the US.

### Patent certification

According to the patents listed by the branded drug applicant in the patent register, the generic drug applicant must address each patent by certifying a patent when it submits an ANDA with one of four types of certification, referred to as Paragraphs 1 (P1), 2 (P2), 3 (P3), or 4 (P4) certifications [9]. South Korea divides patent certifications into five types [10]. Compared with the U.S., China exhibits differences in terms of P2 and P4. China’s P2 is subdivided into three types, namely, patent termination, invalidation, and licensing, while in the U.S., P2 denotes that the listed patent has expired (including natural termination and invalidation) [11]. P4 in China is subdivided into P4.1, in which the listed patent is invalid, and P4.2, in which the generic drug does not fall within the scope of the listed patent rights protection. Among these subtypes, P4.2 in China is different from that in the U.S. (the listed patent will not be infringed upon), mainly because the Bolar exemption clause in China patent law does not consider the submission of an ANDA an infringement [12]. Therefore, in the ANDA review stage, the patentee is not qualified to litigate.

### Approval of ANDA

For ANDAs (P3 in the U.S., P3 in China, and P2 in South Korea), which need to wait for the patent to expire before marketing, the approval results are different. In the U.S., an ANDA with P3 will be granted tentative approval after the completion of technical review, which means that it is not an approved drug until the U.S. Food and Drug Administration (FDA) issues an approval letter after patent expiration [13]. In China, there is no tentative approval; an ANDA with P3 will be formally approved after the technical review is completed, but the generic manufacturer needs to promise that the generic drug will not be marketed before the expiration of the patent, thus exercising self-restraint behavior.

### Procedure linkage of the patent challenge

The submission of P4 (China/U.S.) or P5 (South Korea) by generic drug applicants represents a patent challenge for the patentee but differs in terms of procedural requirements. First, the U.S. and South Korea have specific requirements for the notification of generic drug applicants (for 20 days) [9], but China currently stipulates only that applicants need to notify the holder of the reference drug and keep that record on file. In addition, the CDE should disclose the application information and corresponding certification on the Registration Platform within 10 working days after ANDA acceptance. This difference will also remind the holder of the reference drug to pay attention to ANDA in a timely manner. Second, the length of the stay period varies from 9 months in China and South Korea to 30 months in the U.S. In addition, the stay of ANDA marketing approval in South Korea requires an application review process [9], while such a stay can be automatically obtained after the patentee files a lawsuit in both China and the U.S.

### First generic exclusivity

It is common practice for exclusivity to be granted to the first generic drug with successful patent challenges, but the specific rules vary. First, in the applicable situation, the first generic applicant(s) to provide P4 certification could be eligible for exclusivity in the U.S [14].. However, China is limited to the first generic drug that has submitted to P4.1 certification. In contrast, the scope of first generic exclusivity in China is narrow, which is more conducive to public access to medicines. Second, in terms of the exclusivity duration, China has the longest duration (12 months), South Korea has the second longest duration (9 months), and the U.S. has the shortest duration (180 days). However, China stipulates that such exclusivity period shall not exceed the original patent term of the challenged patent and from the date of approval of the generic drug.

Patent listing and patent certification are two important indicators reflecting the implementation of the patent linkage; these indicators can not only reveal the enthusiasm and characteristics of the innovator’s patent registration but also reveal the actual situation of ANDAs. Thus, the impact of system implementation on the development of generic drugs and public access to medicines can be analyzed. Therefore, data retrieval and analysis to determine the outcomes and characteristics of China’s patent linkage system are conducted in this study.

## Method

### Study design

This was a cross-sectional study of patent listing and patent certification in China after the introduction of the patent linkage system. The data were collected through June 30, 2023. Because China’s patent linkage system is currently implemented only for chemical drugs, this study analyzed only the statistics of the relevant chemical drug data. The time of patent registration and the number of patents were used to present the patent listing behavior of branded drugs. Furthermore, in this study, ANDAs received by the NMPA between July 4, 2021, and June 30, 2023, were retrieved and analyzed according to four types of patent certifications to determine the possible impact of the system on generic drug development and public access to medicines.

### Data source

All the data in this study are from the Registration Platform, which is publicly accessible and searchable on the CDE website [15]. The platform includes three modules: Patent Registration, Patent Information Publicity, and Patent Certification. The last two modules were used in this study. Since some branded drugs already had patents before the implementation of the patent linkage, patent preregistration was conducted on the Registration Platform before official operations began, and branded drug manufacturers were allowed to confirm and disclose patents during the period from June 25 to July 3, 2021 [16]. After the official operation of the Registration Platform began on July 4, 2021, patent listings and certifications were updated daily. Therefore, the patent listing data used in this study were collected from June 25, 2021, to June 30, 2023, and the patent certification data were collected from July 4, 2021, to June 30, 2023. For patent listing, we collected information on the drug name, drug approval number, dosage form, strength, marketing authorization holder (MAH), first registration time of the patent, number of patents, and patent status [17]. For patent certification, we collected information on the drug name, ANDA acceptance number, registration classification of ANDA, ANDA applicant, approval number of the reference drug, MAH of the reference drug, type of patent certification, and publication date of patent certification [18]. Moreover, for P2, we included details on the cases that were terminated, invalidated, and licensed. For P3, we further searched the corresponding patent expiration date and then calculated the remaining level of patent validity between the filing date of ANDA and the expiration date of the patent.

### Analytical methods

Due to the lack of official reviews and duplication limitation functions of the Registration Platform, duplications in both the publications of listed patents and patent certifications were present. Therefore, in this study, we deduplicated the two kinds of original data to improve their accuracy for the data analysis. For patent listing, the statistical items included the year of patent registration, year of drug approval, MAH type, administration, patent status, and number of listed patents. The application of chemical generic drugs in China involves three registration classifications, namely, Class 3 (the domestic applicant imitates the branded drug listed abroad but not listed in China), Class 4 (the domestic applicant imitates the branded drug that has been listed in China), and Class 5.2 (the generic drugs listed abroad apply for listing in China). The situation of the patent certifications of different registered classifications can reflect the status of generic drug R&D and provide reference information for branded drugs with different listing statuses. Therefore, for patent certification, the statistical items included the annual analysis of the number of different certifications from P1 to P4, the P2 and P4 types, the remaining validity of the patent corresponding to the P3, and the corresponding registration classification of different certifications. Microsoft Excel 2016 was used to calculate the descriptive statistics.

## Results

### Patent listing of branded drugs

Before the implementation of the patent linkage system, China registered and publicized the existing patents for branded drugs. Therefore, to distinguish the situation of stock registration, this study divided 2021 into two parts—2021 (before July 4) and 2021 (after July 4). Among them, 2021 (after July 4) contains the information registered on July 4.

For patent listing, the original data of 802 drugs were obtained, and after duplicates were removed according to the approval number, the patents of a total of 632 drugs were listed on the Registration Platform (Table 1). Of these 632 drugs, 462 (73.1%) drugs were approved before the year of registration (stock registration type), and 170 (26.9%) drugs were approved in the year of registration (incremental registration type) when analyzed at the time of drug approval. According to the annual distribution, the number of stock registrations decreased from 233 (91%) in 2021 (before July 4) to 32 (42.7%) in 2023, and the number of incremental registrations increased from 23 (9%) in 2021 (before July 4) to 43 (57.3%) in 2023. According to MAH type, in total, there were 296 domestic manufacturers (46.8%) and slightly more foreign manufacturers, at 336 (53.2%); however, in 2021 (before July 4), foreign manufacturers had an obvious advantage, totaling up to 181 (70.7%). According to the route of administration, oral administration accounted for a large proportion of both the total and annual amounts. According to the legal status of the registered patents, 594 (94%) drug patents were valid, 17 (2.7%) drug patents were partially valid, and 21 (3.3%) drug patents were terminated or invalidated.

**Table 1:** Characteristics of the patent listings for branded drugs sorted by registration year

Registration year	2021 (Before July 4)	2021 (After July 4)	2022	2023 (First half)	Total
Number of drugs (N)	256	204	97	75	632
Drug approval time, no. (%)					
In registration year	23(9)	50(24.5)	54(55.7)	43(57.3)	170(26.9)
Prior to registration year	233(91)	154(75.5)	43(44.3)	32(42.7)	462(73.1)
MAH type, no. (%)					
Foreign	181(70.7)	79(38.7)	50(51.5)	26(34.7)	336(53.2)
Domestic	75(29.3)	125(61.3)	47(48.5)	49(65.3)	296(46.8)
Administration, no. (%)					
Oral	215(84)	125(61.3)	61(62.9)	51(68)	452(71.5)
Injection	23(9)	60(29.4)	31(32)	20(26.7)	134(21.2)
Others	18(7)	19(9.3)	5(5.1)	4(5.3)	46(7.3)
Legal status of patent, no. (%)					
Valid	235(91.8)	192(94.1)	92(94.8)	75(100)	594(94)
Partially valid	8(3.1)	9(4.4)	0(0)	0(0)	17(2.7)
Terminated or invalidated	13(5.1)	3(1.5)	5(5.2)	0(0)	21(3.3)

Table 2 presents the number of patents listed on the Registration Platform per drug sorted by registration year. Of these 632 drugs, the mean value was 1.82, which ranged from 2.21 in 2021(before July 4) to 1.64 in 2023. During the observation period, the mean number of patents for drugs approved before the patent registration year was 1.87, while that for drugs approved during the patent registration year was 1.69. Interestingly, drugs introduced by foreign manufacturers had more patents than did drugs introduced by domestic manufacturers (2 versus 1.61, respectively).

**Table 2:** Number of patents listed on the Registration Platform per drug sorted by registration year

Registration year	2021 (Before July 4)	2021 (After July 4)	2022	2023 (First half)	Total
Number of drugs (N)	256	204	97	75	632
Mean	2.21	1.55	1.48	1.64	1.82
Standard deviation	1.74	1.05	0.79	1.27	1.40
Drug approval time					
In registration year	1.83	1.68	1.7	1.6	1.69
Prior to registration year	2.14	1.51	1.21	1.69	1.87
MAH type					
Foreign	2.29	1.54	1.74	1.85	2
Domestic	1.6	1.56	1.21	1.53	1.61
Administration					
Oral	2.26	1.58	1.54	1.61	1.9
Injection	1.61	1.53	1.29	1.65	1.51
Others	2.5	1.42	2	2	1.96

### Patent certification of ANDAs

For patent certification, the original data of 5251 ANDAs were obtained, and after duplicates were removed according to the ANDA acceptance number, a total of 5058 ANDAs were included in the study (Table 3). Among the 5058 ANDAs, P1 accounted for the majority (4303, 85.1%), indicating that ANDAs were still dominated by the imitation of reference drugs without patent listings. P3 had the second highest percentage (6.1%), while P2 and P4 had similar percentages (4.5% versus 4.3%, respectively). We analyzed characteristics by year and registration classification. Interestingly, in the annual analysis, we found that the annual proportion of P2 certifications increased from 2.7% in 2021 to 5.1% in 2023. However, the annual proportions of P3 and P4 certifications both decreased, with P3 certifications decreasing from 7.5% in 2021 to 5.1% in 2023 and P4 certifications decreasing from 5.1% in 2021 to 3.5% in 2023. In addition, 98.7% of class 3 applications are P1, 83.6% of class 5.2 applications are P1, and only 77.1% of class 4 applications are P1 given the fact that patent registration is limited to drugs marketed in China.

**Table 3:** Outcomes of the patent certification of ANDAs in China

	P1	P2	P3	P4	Total
ANDA, no. (%)	4303(85.1)	226(4.5)	309(6.1)	220(4.3)	5058
Year					
2021 (Second half), no. (%)	809(84.7)	26(2.7)	71(7.5)	49(5.1)	955
2022, no. (%)	1905(84.3)	105(4.6)	144(6.4)	107(4.7)	2261
2023 (First half), no. (%)	1589(86.3)	95(5.1)	94(5.1)	64(3.5)	1842
Registration classification					
Class 3, no. (%)	1775(98.7)	2(0.1)	10(0.6)	11(0.6)	1798
Class 4, no. (%)	2350(77.1)	210(6.9)	291(9.6)	196(6.4)	3047
Class 5.2, no. (%)	178(83.6)	14(6.6)	8(3.8)	13(6.1)	213

**Notes:**

P1: the number of ANDAs that submitted only P1 certification(s)

P2: the number of ANDAs that submitted only P2 certification(s)

P3: the number of ANDAs that submitted only P3 certification(s) and the number of ANDAs that submitted P3 and P2 certifications

P4: the number of ANDAs that submitted only P4 certification(s) and the number of ANDAs that submitted P4 and P2 and/or P3 certifications

Table 4 presents the outcomes of the ANDAs that were submitted for P2 or P4 certifications in detail. A total of 310 ANDAs were submitted for P2, including 226 ANDAs for P2 only and 84 ANDAs for “P2 and P3 and/or P4”. In China, P2 is subdivided into three types: patent termination, invalidation, and licensing. Interestingly, only 47.1% of the 310 ANDAs were terminated, while 46.4% were invalidated. This figure is even more prominent in “only P2”, where 58.8% of the ANDAs were invalidated. From the annual trend, the total number of P4 certifications shows a steady and slightly increasing trend, from 49 in 2021 (half a year) and to 107 in 2022 and then to 64 in 2023 (half a year). In terms of their respective proportions, among 220 ANDAs, P4.2 reached 77.7%, significantly more than P4.1 (17.3%), and the annual proportion of P4.2 showed an upward trend, increasing from 69.4% in 2021 to 82.8% in 2023.

**Table 4:** Outcomes of ANDAs that were submitted for P2 or P4 certifications

	2021 (Second half)	2022	2023 (First half)	Total
**Total number of P2 (N)**	36	149	125	310
Patent termination, no. (%)	6(16.7)	76(51)	64(51.2)	146(47.1)
Patent invalidation, no. (%)	25(69.4)	62(41.6)	57(45.6)	144(46.4)
Patent licensing, no. (%)	0(0)	3(2)	4(3.2)	7(2.3)
Invalidation and licensing, no. (%)	3(8.3)	2(1.4)	0(0)	5(1.6)
Termination and invalidation, no. (%)	2(5.6)	6(4)	0(0)	8(2.6)
**Only P2 (N)**	26	105	95	226
Patent termination, no. (%)	3(11.5)	40(38.1)	41(43.2)	84(37.2)
Patent invalidation, no. (%)	23(88.5)	60(57.1)	50(52.6)	133(58.8)
Patent licensing, no. (%)	0(0)	3(2.9)	4(4.2)	7(3.1)
Invalidation and licensing, no. (%)	0(0)	2(1.9)	0(0)	2(0.9)
**P2 and P3 and/or P4 (N)**	10	44	30	84
Patent termination, no. (%)	3(30)	36(81.8)	23(76.7)	62(73.8)
Patent invalidation, no. (%)	2(20)	2(4.6)	7(23.3)	11(13.1)
Termination and invalidation, no. (%)	2(20)	6(13.6)	0(0)	8(9.5)
Invalidation and licensing, no. (%)	3(30)	0(0)	0(0)	3(3.6)
**Total number of P4 (N)**	49	107	64	220
**P4.1, no. (%)**	15(30.6)	16(15)	7(10.9)	38(17.3)
**P4.2, no. (%)**	34(69.4)	84(78.5)	53(82.8)	171(77.7)
**P4.1 and P4.2, no. (%)**	0(0)	7(6.5)	4(6.3)	11(5)

Table 5 describes the remaining validity of the patents corresponding to P3 certification. A total of 390 ANDAs were included in this section, including “only P3” and “P3 combined with other patent certifications”. Considering the influence of extreme values on the remaining patent validity, the median value was used in the analysis, and the interquartile range (IQR) and the range of the median value were calculated. Among the 390 ANDAs observed, the median duration of patent remaining validity was 17 months, ranging from − 2 to 204 months. Interestingly, this value was greater in “P3 and P2”, with a median of 45 months, than in the other certifications.

**Table 5:** Remaining validity of the patent corresponding to P3 certification (month)

	2021 (Second half)	2022	2023 (First half)	Total
Total number of P3 (N)	90	184	116	390
Median (IQR)[range], month	19.5(15–32)[5–92]	18(9–29)[-1-95]	14(8-26.25)[-2-204]	17(10-30.75)[-2-204]
Only P3 (N)	65	129	86	280
Median (IQR)[range], month	17(15–31)[5–92]	15(9–27)[-1-95]	14(8–27)[-2-204]	16(9.75–29.25)[-2-204]
P3 and P2 (N)	6	15	8	29
Median (IQR)[range], month	60.5(59–62)[14–62]	21(12-48.5)[7–58]	44.5(12-45.25)[-1-46]	45(12–49)[-1-62]
P3 and P4 (N)	19	24	14	57
Median (IQR)[range], month	22(19–29)[10–75]	14(4.5–64)[1–67]	2(1.25–13.75)[0–23]	15(5–27)[0–75]
P3 and P2 and P4 (N)	0	16	8	24
Median (IQR)[range], month	NA	23(19.5-25.25)[18–34]	19(17–22) [13–22]	21(18–25)[13–34]

Note: when an ANDA submitted more than one P3 certifications, the remaining patent validity was measured according to the latest expired patent

NA: not applicable

## Discussion

### China’s system aims to promote the balance of multiple interests—innovation, imitation and public health

Unlike other drug patent systems, patent linkage is considered a “nonzero-sum” game involving multiple interests and that can achieve multiwin policy effects [19]. On the one hand, this linkage provides innovators with the early warning and prevention of generic drug marketing through patent listing and safeguards innovation rights and interests. On the other hand, this linkage can reduce the degree of patent infringement risk of generic drugs after marketing and stimulate patent challenges, which is conducive to the development of high-quality generics and the overall improvement in drug accessibility in the long term, to achieve a balance among innovation, imitation and public drug accessibility. Given the characteristics of China’s system, public access to medicines is obviously the first consideration. Generally, there are two reasons for worrying about the impact of patent linkage on drug accessibility: first, the term of stay will prolong the review period of ANDAs, thus delaying the marketing of generic drugs, and second, first generic exclusivity will undoubtedly lead to a monopoly on the basis of the patent period, thus undermining public health benefits.

In this regard, China has made effective arrangements when setting up the system. First, the stay period is set to 9 months, which is significantly less than the 30 months in the U.S., and the statutory review time limit for ANDA is 200 working days [20], which is converted into a natural day, that is, more than 9 months. Therefore, even if there is no stay period, the normal ANDA review time will be more than 9 months, and thus, the stay period will not extend the ANDA review period. According to the 2022 Annual Drug Evaluation Report, the on-time completion rate of ANDAs increased from 76.8% in 2020 to 95.7% in 2021 and 99.45% in 2022 [21]. That is, the vast majority of the reviews were completed within the statutory time limit (more than 9 months), and there was no major lag due to the stay period. Second, China’s first generic exclusivity is used only for the case of submitting for P4.1 certification, and such exclusivity should not exceed the original patent period of the challenged patent; thus, a monopoly will not be added on the basis of the original patent period. An analysis of the specific types of P4 certification can better illuminate this point. Among these types, P4.1 accounts for 17.3% and will obtain first generic exclusivity after approval; however, it will not exceed the original patent period of its challenge and, thus, will not have an impact on the original expectation of public drug accessibility. The proportion of P4.2 certifications is greater than that of P4.1 certifications, reaching 77.7%. First generic exclusivity is not obtained after approval, and thus, this approach not only affects the original expectation of drug accessibility but also breaks the patent monopoly of the branded drug, provides the public with additional choices, and has a positive effect on the accessibility of medicines to the Chinese public.

### Branded drug manufacturers should pay attention to the influence of patent linkages

From the above results, it can be seen that in terms of the patent listings of branded drugs, stock registration has been declining, and incremental registration has gradually increased and come to the forefront (57.3%). This result shows that in the past three years following the implementation of the system, stock registration has been gradually completed, and incremental registration may become mainstream in the future. According to the MAH type analysis, foreign manufacturers have a slightly higher number of drugs listed their patents (53.2% versus 46.8%, respectively) and mean number of patents per drug (2% versus 1.61%, respectively) than do domestic manufacturers. However, combined with annual analysis and patent certifications, branded drug manufacturers should pay attention to the below two points.

First, foreign manufacturers account for a large proportion of stock registrations, but their proportion of incremental registrations is shrinking; for example, in 2021 (before July 4), which is dominated by stock registration, foreign manufacturers have an obvious advantage, up to 181 (70.7%), but in 2023, which is dominated by incremental registration, foreign manufacturers account for only 34.7%. The reason for this is due mainly to the fact that China’s patent list registers only drugs marketed in China, and thus, some drugs listed abroad are delayed in terms of patent registration due to their failure to enter China in time. This delay is conducive to the development of class 3 applications and an important reason why the proportion of P1 in class 3 applications is as high as 98.7%. Therefore, foreign manufacturers of branded drugs that have been listed abroad but not yet listed in China should pay attention to the influence of patent linkages and establish a layout in advance. On the one hand, these branded drugs should enter the Chinese market as early as possible so that patents can be registered in time and patent disputes can be resolved before generic drugs are marketed. On the other hand, these manufacturers should pay attention to the approval of ANDAs before branded drugs are listed in China to initiate timely patent infringement lawsuits. Unlike patent linkage, these patent infringement lawsuits cannot be filed until the generic drug is marketed.

Second, branded drug manufacturers should pay attention to the premature submission of ANDA for P3 certification. The remaining validity of the patent corresponding to P3 shows how long before the expiration of the relevant patent the generic drug manufacturer submits ANDA and its impact on the branded drug. According to the remaining validity of the patent corresponding to P3 described in Table 5, the median value reached 17 months, and the highest reached 240 months. The median value of ANDAs submitted for P3 and P2 certifications is greater, reaching 45 months, which means that generic drug manufacturers submit ANDAs 3–4 years before the expiration of the corresponding patents. This situation would not have an impact on the branded drugs, but China’s approval of an ANDA with P3 certification does not have “tentative approval”, similar to that of the U.S. An ANDA will be formally approved after the completion of a technical review, but the approved generic manufacturer needs to promise that the generic drug will not be marketed before the expiration of the patent. This self-restraint behavior of the generic manufacturer will obviously destabilize the interests of patentees, and the branded drug manufacturer (or patentee) will face potential infringement risks.

### Generic drug manufacturers carried out patent challenges earlier, but the growth was not obvious, and most of them were P4.2

The analysis results for P2 show that 46.4% of the patents were invalidated (Table 4), which indicates that before the implementation of the patent linkage system, generic drug manufacturers launched patent challenges, and after the implementation of the system, the patents were judged to be invalid, after which ANDAs with P2 certification were filed. The reason for this is mainly that in October 2017, the General Office of the Communist Party of China Central Committee and the General Office of the State Council jointly issued the *Opinions on Deepening the Reform of the Review and Approval System and Encouraging the Innovation of Drugs and Medical Devices*, which included a clear proposition to “explore and establish a drug patent linkage system” [22]. This policy document released the signal that China would establish a patent linkage. Therefore, some generic drug manufacturers initiated patent challenges and carried out corresponding patent invalidity lawsuits before the formal implementation of the system.

After the formal implementation of the patent linkage system, the growth in the number of P4 certifications was not obvious, accounting for only approximately 4%. Among these, P4.2 certifications accounted for a larger proportion, reaching 77.7%, and showed an increasing trend (from 69.4% in 2021 to 82.8% in 2023). This finding shows that generic drug manufacturers are increasingly inclined to file a lawsuit that does not fall within the scope of patent protection, which is less difficult than is a patent invalidity lawsuit; however, it is worth noting that P4.2 certifications do not obtain first generic exclusivity, and only P4.1 certifications are eligible for such exclusivity. In addition, among the 178 class 5.2 ANDAs (foreign generic drug applications), P1 certifications still dominates, reaching 83.6%, while P4 certifications accounts for 6.1%, indicating that foreign companies have also begun to launch patent challenges in China. Therefore, foreign generic drug applicants also need to be familiar with the details of China’s patent linkage system, especially the difference between P4.1 and P4.2 certification, to seek the most favorable type of patent certification.

### Study limitations

This study has several limitations. First, since the system has been implemented only for a short time, the statistical duration of this study was shorter than that of the other studies, and thus, the analysis of outcomes and impacts would be more convincing if the observation time were extended. Second, the statistical sample “drug” in this study was calculated according to the approval number, and the “ANDA” was calculated according to the acceptance number. Both of these terms refer to the same manufacturer, the same active ingredient, the same dosage form, and the same strength. For example, 10-mg or 5-mg dapagliflozin tablets are considered different drugs or ANDAs, but in most cases, the patents listed or patent certifications submitted for different product strengths are the same; therefore, this statistical caliber also limits this study. We will conduct a single-variety (combining different specifications into one) analysis in future studies.

## Conclusions

China’s patent linkage system aims to promote the balance of multiple interests, such as innovation, imitation, and public health. Therefore, compared with the systems in the U.S. and South Korea, more localized adjustments have been made in system design, revealing its own characteristics, in China. Patent listings and certifications are the key indicators that reflect the balance of these benefits after the implementation of the system. From the perspective of system outcomes, patent registration is gradually shifting from stock registration to incremental registration, which will effectively safeguard innovators’ rights and interests. Moreover, foreign branded drug manufacturers need to pay attention to the impact of the system. ANDAs have been connected to the patent linkage system in an orderly manner, and the number of patent challenges has not increased significantly. However, with the accumulation of patent challenge experience, this system can also promote the high-quality development of China’s generic drugs in the future. The pursuit of public health welfare requires both the maintenance of innovation to continue providing more effective drugs and the encouragement of imitation to provide more affordable drugs. Therefore, against the background of promoting the balanced development of innovation and imitation, under the specific design of the system, patent linkage will also have a positive effect on the accessibility of medicines to the Chinese public.

### Electronic supplementary material

Below is the link to the electronic supplementary material.

**Additional file 1**. Comparison of patent linkage systems in China, the U.S. and South Korea.

## Data Availability

The data underlying this article will be shared upon reasonable request to the corresponding author.

## Abbreviations

ANDA: Abbreviated new drug application (also known as generic drug application)
CNIPA: China National Intellectual Property Administration
CDE: Center for Drug Evaluation
FDA: U.S. Food and Drug Administration
MFDS: Ministry of Food and Drug Safety
MAH: Marketing authorization holder
NMPA: National Medical Products Administration
