# Supplementary material for: Characteristics and outcomes of the drug patent linkage system in China
Source: Global Health. 2024 Apr 15;20:31. doi: 10.1186/s12992-024-01035-x (PMC11017475; doi:10.1186/s12992-024-01035-x)
Supplement: Supplementary file 1 — Additional file 1. Comparison of patent linkage systems in China, the U.S. and South Korea. [file 12992_2024_1035_MOESM1_ESM.docx]

**Additional file 1.**

**Table S1 Comparison of patent linkage systems in China, the U.S. and South Korea**

|  | **the US** | **South Korea** | **China** |
| --- | --- | --- | --- |
| **Scope of application** | Chemicals | Chemicals and Biologics | Chemicals (complete implementation) |
| **Patent listing** | Patent list: Orange Book  Patents that may be listed：  (1) drug substance  (2) drug product  (composition and formulation)  (3) pharmaceutical use  Timing of submission of patent information: With NDA or within 30 days of patent grant  Management of the list: FDA, with administrative Process  Amendments to the list: Possible, by the NDA applicant | Patent list: Green List  Patents that may be listed：  (1) drug substance  (2) composition  (3) dosage form  (4) pharmaceutical use  Timing of submission of patent information: Within 30 days of the date of MA or patent grant  Management of the list: MFDS, with effective examination  Amendments to the list: Possible, by the NDA applicant or MFDS | Patent list: China's Patent Information Registration Platform for Marketed Drugs  Patents that may be listed：  (1) drug substance  (2) composition  (3) pharmaceutical use  Timing of submission of patent information: Within 30 days of the date of MA or patent grant  Management of the list: CDE, with administrative process  Amendments to the list: Possible, by the NDA applicant |
| **Patent certification** | P1- no patent is listed  P2- the listed patent has expired  P3- the listed patent will expire before approval is granted  P4- the listed patent is invalid, unenforceable, or will not be infringed | P1- the listed patent has expired  P2- the applicant will not market the generic drug until the listed patent expires  P3- the patentee or the holder of the reference drug consent to not giving patent notice  P4- the scope of listed patent does not cover the pharmaceutical use of the ANDA  P5- the listed patent is invalid, or will not be infringed | P1- no patent is listed  P2- the listed patent has been terminated or declared invalid, or the applicant for the generic drug has been granted a patent license  P3- the generic drug applicant undertakes not to put the generic drug on the market until the listed patent expires  P4- the listed patent is invalid, or the generic drug does not fall within the scope of the listed patent right protection |
| **Links to procedures for patent challenges** | Timing of notification: within 20 days after ANDA is filed  Timing of request: Within 45 days after the date of receipt for notification  Stay of generic market approval: 30 months | Timing of notification: within 20 days after ANDA is filed  Timing of request: Within 45 days after the date of receipt for notification  Stay of generic market approval: 9 months | Timing of notification: Not regulated  Timing of request: Within 45 days after the date of publication of ANDA  Stay of generic market approval: 9 months |
| **Approval of ANDA** | P1-2: Approval  P3: Tentative approval  P4: Depending on the patent litigation | P1\3\4: Approval  P2: Approval, but cannot be marketed until the list patent expire  P5: Depending on the patent litigation | P1-2: Approval  P3: Approval, the generic drug applicant undertakes not to put the generic drug on the market until the listed patent expires  P4: Depending on the patent litigation |
| **First generic exclusivity** | Requirements for exclusivity: The first ANDA containing P4 certification  Exclusivity period: 180 days  A starting point of the exclusivity：the date of the first commercial marketing | Requirements for exclusivity: (1) the first ANDA that has challenged a patent and obtained a favorable decision; or  (2) the first ANDA that has filed a challenge within 14 days of the first challenge and has become the first to obtain a favorable decision  Exclusivity period: 9 months, additional 2-month period is available to compensate for delayed marketing due to the reimbursement process  A starting point of the exclusivity：the date of the marketing | Requirements for exclusivity: The first ANDA containing P4.1 certification  Exclusivity period: 12 months, but not exceeding the original patent term of the challenged patent.  A starting point of the exclusivity：the date of approval |
